# Supplementary material for: Association between Dietary Diversity Score and Metabolic Syndrome in Korean Adults: A Community-Based Prospective Cohort Study
Source: Nutrients. 2022 Dec 13;14(24):5298. doi: 10.3390/nu14245298 (PMC9784032; doi:10.3390/nu14245298)
Supplement: Supplementary file 1 [file nutrients-14-05298-s001.zip › nutrients-2066609-supplementary.pdf]

Supplementary Table S1. Classification of food items.

5 Major food groups		Food items
Grains	Staple foods made from grains	white rice, white rice cake/rice-cake soup, steamed white rice cake/injeolmi, wheat noodles, jajangmyeon, naengmyeon/buckwheat noodles, powder of roast grain, dumplings, bread, red bean bread/steamed bun, other bread, corn flakes, snacks, muk, cellophane noodles
	Whole grains	rice with barley, rice with other cereals
	White roots/tubers	potato, sweet potato
	Instant noodles	ramyon
	Fast food	pizza/hamburger
Meat, fish, eggs, and beans	Processed meat	ham/sausages
	Processed red meat (ruminant)	roasted beef, beef stew, galbijjim, dog meat, organs from animals
	Processed red meat (non-ruminant)	grilled pork belly, roasted pork, steamed pork
	Poultry	chicken
	Fish & seafood	sashimi, hairtail, ell, croaker, pollack, frozen pollack, dried pollack, blue-backed fish, anchovy, squid, small octopus, canned tuna, fish cake/crab meat, crab, clam, oyster, shrimp, salted seafood
	Eggs	Eggs, quail eggs
	Legumes	beans/beans cooked in soy sauce, bean paste soup, tofu, soybean milk
	Nuts & seeds	peanuts/almonds/pine nut
Vegetables	Vitamin A-rich orange vegetables	carrot/carrot juice, pumpkin, zucchini
	Dark green leafy vegetables	pepper leaves, sesame leaf, spinach, chives/water celery, other green vegetables

	Other vegetables	cabbage, cabbage kimchi, sweet potato vines, bean sprouts, deodeok/balloon flower, radish, radish kimchi, radish water kimchi, other kimchi, pickled vegetables onion, pepper, lettuce, mushroom, other mushrooms, cucumber, seaweed, tomato/tomato juice, vegetable juice, green juice
Fruits	Vitamin A-rich fruits	persimmon
	Citrus	orange/orange juice, tangerine
	Other fruits	strawberry, oriental melon/watermelon, banana, pear, peach/plum, apple/apple juice, grape/grape juice
	Fruit juice	orange/orange juice, apple/apple juice, grape/grape juice
Milk	Cheese	cheese
	Yogurt	yogurt
	Fluid milk	milk

Supplementary Table S2. Changes in body weight and metabolic syndrome components during follow-up among men.

	Men								
	≤3 (*n* = 524)		*P*-value	4 (*n* = 1,156)		*P*-value	5 (*n* = 1,144)		*P*-value
	**At baseline**	**At follow-up**		**At baseline**	**At follow-up**		**At baseline**	**At follow-up**	
Weight (kg)	64.5 ± 0.39	65.5 ± 0.52	0.8263	64.5 ± 0.26	64.7 ± 0.35	0.0121	66.0 ± 0.26	66.6 ± 0.35	0.5193
Abdominal obesity (cm)	81.2 ± 0.29	86.2 ± 0.45	<.0001	80.8 ± 0.19	84.8 ± 0.30	<.0001	81.4 ± 0.20	85.3 ± 0.30	<.0001
Elevated blood pressure (mmHg)									
SBP	119.5 ± 0.65	120.4 ± 0.84	0.3844	117.9 ± 0.44	119.0 ± 0.56	0.0001	117.0 ± 0.44	118.7 ± 0.55	0.0012
DBP	80.3 ± 0.44	77.7 ± 0.55	0.0001	79.4 ± 0.30	76.9 ± 0.37	0.0008	78.9 ± 0.29	77.6 ± 0.36	0.0002
Hypertriglyceridemia (mg/dL)	157.6 ± 4.18	139.2 ± 5.68	0.0053	150.9 ± 2.82	135.5 ± 3.83	0.0002	145.8 ± 2.83	136.0 ± 3.77	0.0056
Elevated fasting glucose (mg/dL)	84.7 ± 0.58	94.4 ± 0.87	<.0001	84.9 ± 0.39	95.4 ± 0.58	<.0001	85.7 ± 0.39	94.5 ± 0.57	<.0001
Reduced HDL cholesterol (mg/dL)	45.7 ± 0.43	45.7 ± 0.67	0.8287	45.5 ± 0.29	45.0 ± 0.45	0.9641	45.6 ± 0.29	46.0 ± 0.44	0.0458

Supplementary Table S3. Changes in body weight and metabolic syndrome components during follow-up among women.

	Women					
	≤3 (*n* = 297)	*P*-value	4 (*n* = 925)	*P*-value	5 (*n* = 1,422)	*P*-value

	At baseline	At follow-up		At baseline	At follow-up		At baseline	At follow-up	
Weight (kg)	56.0 ± 0.46	56.3 ± 0.60	0.1475	56.4 ± 0.26	56.1 ± 0.34	0.0273	57.2 ± 0.21	57.4 ± 0.27	0.1616
Abdominal obesity (cm)	78.6 ± 0.48	84.7 ± 0.65	<.0001	77.3 ± 0.27	82.0 ± 0.38	<.0001	76.6 ± 0.21	81.6 ± 0.29	<.0001
Elevated blood pressure (mmHg)									
SBP	113.9 ± 0.89	119.1 ± 1.20	0.0002	113.0 ± 0.50	115.3 ± 0.70	<.0001	111.8 ± 0.41	115.7 ± 0.54	<.0001
DBP	76.0 ± 0.58	74.8 ± 0.71	0.0600	74.5 ± 0.33	73.3 ± 0.41	0.5211	73.9 ± 0.27	73.9 ± 0.32	0.2731
Hypertriglyceridemia (mg/dL)	114.0 ± 2.90	124.2 ± 5.02	0.0922	114.4 ± 1.64	118.0 ± 2.92	0.0979	112.7 ± 1.32	114.8 ± 2.27	0.3850
Elevated fasting glucose (mg/dL)	82.2 ± 0.60	92.9 ± 1.08	<.0001	80.5 ± 0.34	90.2 ± 0.63	<.0001	80.8 ± 0.27	90.8 ± 0.49	<.0001
Reduced HDL cholesterol (mg/dL)	48.3 ± 0.58	49.1 ± 0.86	0.6150	48.8 ± 0.33	50.4 ± 0.50	<.0001	48.8 ± 0.26	50.6 ± 0.39	<.0001
